# Popper’s Critical Rationalism as a Response to the Problem of Induction: Predictive Reasoning in the Early Stages of the Covid-19 Epidemic

**DOI:** 10.1007/s40926-022-00203-6

**Published:** 2022-10-24

**Authors:** Tuomo Peltonen

**Affiliations:** Aalto University Business School, Espoo, Finland

## Abstract

The extent of harm and suffering caused by the coronavirus pandemic has prompted a debate about whether the epidemic could have been contained, had the gravity of the crisis been predicted earlier. In this paper, the philosophical debate on predictive reasoning is framed by Hume’s problem of induction. Hume argued that it is rationally unjustified to move from the finite observations of past incidences to the predictions of future events. Philosophy has offered two major responses to the problem of induction: the pragmatic induction of Peirce and the critical rationalism of Popper. It is argued that of these two, Popper’s critical rationalism provides a more potent tool for preparing for unanticipated events such as the Covid-19 pandemic. Popper’s notion of risky predictions equips strategic foresight with clear hypotheticals regarding potential crisis scenarios. Peirce’s pragmatic induction, instead, leans on probabilities that are slower to be amended as unexpected events start unfolding. The difference between the two approaches is demonstrated through a case study of the patterns of reasoning within the World Health Organization in the early stages of the coronavirus pandemic.

**Supplementary information:**

The online version contains supplementary material available at 10.1007/s40926-022-00203-6.

## Introduction: Covid-19 and Predictive Reasoning

The Covid-19 pandemic has been the most devastating worldwide biomedical crisis since the Spanish Flu of 1918–1920. By May 2022, the SARS-Cov2-virus has officially infected almost 300 million people worldwide, causing over 6 million deaths (World Health Organization, 2022). The lockdowns, border closures and related mitigating activities have paralyzed the economies, caused a wave of medical and mental problems, and shattered our sense of ontological security.

The extent of harm and suffering caused by the coronavirus pandemic has prompted a debate about whether the epidemic could have been contained, had the gravity of the crisis been predicted earlier (Ioannidis et al. 2020; Taleb et al. 2020; Pinson and Makridakis 2021). At the heart of the controversy is the strategic foresight capability of the World Health Organization, which is the prime institution charged with monitoring and anticipating worldwide biomedical threats and risks (e.g. Cousins 2018; Michelson 2005).

From a philosophy of management point of view, the issue with the potency of organizational risk foresight is related to the more general problem of how it is possible to make justified predictions about future events. A popular answer to the question contends that past observations or experiences can be used as a basis for generalizations that postulate universally valid regularities and patterns. Empirical research or reasoning is often practiced under the assumption that the interpretation of observed instances enables the generation of theories that are universally valid across time (Mill 1843/1906). The method of generalizing from a finite set of observations is thus assumed to lead to justified knowledge about the not-yet-observed future events (Locke 2007). This type of evidence-based predictive reasoning follows the more general form of inductive inferences in management epistemology (Ketokivi and Mantere 2010; Locke 2007).

This paper argues that induction suffers from a fundamental problem and that justified forecasts must rely on other philosophies of reasoning than pure induction. The problem of induction as originally formulated by Hume (1739/2007) casts doubt on our ability to move from the finite observations of past incidences to predictions about the future behavior of a phenomenon. Two prominent responses to problem of induction are discussed in this paper: the pragmatic induction of Peirce (1878) and the critical rationalism of Popper (1971). It is argued that of these two, Popper’s critical rationalism provides a more potent tool for preparing for unanticipated events such as the Covid-19 pandemic. Popper’s notion of risky predictions equips strategic foresight with clear hypotheticals regarding potential crisis scenarios that can be acted upon. Peirce’s pragmatic induction, instead, leans on probabilities that are slower to be amended as unexpected events start unfolding. The difference between the two approaches is demonstrated through a case study of the patterns of reasoning within the World Health Organization in the early stages of the coronavirus pandemic.

The paper is structured as follows. The second section outlines the inductive method of reasoning and presents David Hume’s critique of induction. This is followed by a review of the two main philosophical responses to the problem of induction, namely Peirce’s pragmatic induction and Popper’s critical rationalism. Special attention is paid to the diverging temporal strategies of the two responses. The subsequent two sections present an analysis of the style of predictive reasoning employed in the WHO during the early stages of the pandemic. After a concise overview of the predictive work within the organization, an interpretation of the style of predictive reasoning considering the shortcomings of the pragmatic inductive approach adapted by WHO is presented. The article closes with a concluding segment that summarizes the main argument and discusses the role and contribution of Popperian philosophy in various areas of philosophical management inquiry.

## The Problem of Induction

Generating universally valid theories from empirical observations typically employs the method of inductive reasoning (Ketokivi and Mantere 2010). In inductive reasoning, a set of observations is used to make more general inferences that extend beyond the domain of observed cases or instances. What separates inductive reasoning from other forms of thinking is the logical order of inferences where a reasoner proceeds from observations or experiences through generalization to the postulation of universally valid theories (Tsang and Williams 2012).

However, it has been noted that the weakest element of inductive reasoning is the jump from an analysis of a set of observations to universally valid theories that are presumed to remain true over time. The leap from the observed instances to the unobserved state of future events appears to make assumptions about the continuity of reality that cannot be justified by our observational experiences alone. The validity of inductive reasoning appears to have a major weakness that can compromise the use of induction in warranted predictive reasoning. This is the crux of the so-called problem of induction articulated originally by David Hume (2007; Henderson 2020).

According to Hume (2007), there are two main types of reasoning. Matters of fact draw upon observations of empirical reality to deduce more general patterns and regularities. Relations of ideas, instead, rely on abstract propositions that are logically deduced from given premises. Matters of fact represent inductive reasoning, whose task is to observe a set of events and to infer regularities and causal connections between discreet instances. Relations of ideas, on the other hand, cannot produce new positive knowledge since they rely on abstract analytic reasoning. According to Hume, it is the realm of matters of fact, or induction, which, by applying a synthetic approach to knowledge creation, is responsible for substantive growth in our understanding. The question however is to which extent are our inductive inferences rationally justifiable in a more universal sense of truthfulness?

After some consideration, Hume (2007) concludes that the attempts to justify induction fail in the two main domains. Starting from the deductive justification, he argues that induction cannot corroborate the validity of empirically inferred patterns or generalities since the premises of an inductive argument are not capable of guaranteeing the truthfulness of the conclusions. In the case of predictive arguments based on past observations, there is no logical necessity assuring that the future will resemble the past. Thus, there needs to be a hidden middle premise for the inductive argument to be logically justifiable.

This hidden premise is the assumption that the observed regularity will extend to the unobserved instances as well. In other words, the idea is that the patterns deduced from the observations will apply for every instance, including those events that will take place in the future that has not yet happened. Hume (2007) called this assumption the uniformity of nature. The problem with uniformity of nature is that it cannot be logically justified since from a deductive viewpoint, it is always potentially possible that the past pattern will not be repeated tomorrow. For example, despite our thousands of years of successive observations, there is always a logical possibility that the sun will not rise tomorrow.

Alternatively, the uniformity of nature could be justified by referring to some metaphysical principle about the underlying structure of reality. This was a route that Hume (2007) as an empiricist was inclined to avoid. In any case, even the metaphysical justifications such as the argument for the orderliness of the created universe are unconvincing because they cannot guarantee a potential disruption of the established order. For example, it is conceivable, although not probable, that a distant celestial object moving at the speed of light will hit our planet the very next day. In human and social affairs, metaphysical arguments for the temporal uniformity of reality are equally problematic in light of the recent turns to processual ontologies (e.g. Whitehead, 1927; Habermas 1992).

We therefore often turn to inductive justifications of induction. Theories deduced from generalized observations can be argued to be valid in the future because theories have proven to be accurate predictions in the past. That is, we have evidence that proposed theories or understandings have passed the inductive test since they have demonstrably been shown to predict future events (in the past) (cf. Tetlock and Gardner 2015). For example, the unlikelihood of a global coronavirus pandemic had been seemingly proven by the containment of previous SARS and MERS epidemics (Michelson 2005). The weakness of this justification is that it assumes that the past predictive success of schemes or theories can be used to validate the forecasting potential of those schemes. However, the past success of inductive predictions cannot be taken as the proof that the theories will retain their predictive powers in the not-yet-realized future. This type of justificatory approach appears to lean on a second-order inductive argument. The dilemma here is that the problem of induction cannot be solved by referring to another inductive claim as this type of argumentation leads to circular reasoning. Induction cannot be legitimated by another inductive assertion.

Having weighted both deductive and inductive justifications, Hume (2007) concludes that induction is an inherently irrational form of reasoning. At the same time, he thinks that induction is nevertheless an integral part of our innate everyday life and, as such, vital for the smooth unfolding of routine human actions in different spheres of practice. We use break as we foresee that it causes the car to decelerate. Or managers can believe that a certain measure universally leads to organizational success (March and Sutton 1997). These un-reflexive beliefs are not based on rigorous logical thinking but are, according to Hume, customs of mind formed over time as we experience connections between events. This gradual habituation of our inductive beliefs arises as we experience time and again constant conjectures between things or instances.

In response, Hume thought that inductive reasoning is such an intrinsic part of our cognitive habits that it must have a naturalistic explanation. Inductive imagination based on the exposure to constant conjectures between events or observations might have a grounding in the evolutionary necessities imposed upon the human populations in the distant history. In any case, the apparent linkage of inductive reasoning to our human nature suggests that induction will remain a dominant form of subjective inference in the everyday operations of the human mind.

Taken together, Hume’s (2007) position was that inductive reasoning is rationally unjustifiable but could however be seen as an integral part of our innate human beliefs. Essentially, he argued that we cannot produce justifiable knowledge through inductive inferences, and, without a viable deductive or synthetic alternative, a logical outcome is to adopt the position of philosophical skepticism. There is no rational warrant to inductive predictions.

## Two Philosophical Responses to Hume’s Problem

Hume’s problem has troubled epistemologists and philosophers of science to this day. There have been several attempts at solving the problem Howson 2000; Salmon 1967/2017). It is beyond the limited scope of this article to exhaustively outline and discuss the totality of the proposed solutions. Generally speaking, however, it is possible to identity three kinds of responses to the problem: those that attack the basic elements of Hume’s argument (e.g. Bhaskar, 1975; Bonjour 1986; Black 1958), those that aim to circumvent or deny the validity of the problem (e.g. Strawson 1952; Simon 1973), and those that acknowledge the insolvability of the problem and try to work a way forward given the apparent problems related to inductive reasoning. Insofar as many of the attacks on Hume’s reservations regarding inductive, deductive and nature-uniformist justifications of induction seem to suffer from circulatory or illogical reasoning (Salmon 1953; Johnsen 1972), there are grounds to view Hume’s problem as having survived the majority of challenges. For example, Lange (2011; 44) has recently asserted about the attempts to rebuke Hume’s skepticism that: “[d]espite these massive efforts, no response to date has received widespread acceptance”, while Howson (2000; 10) has noted in his review that the argument of Hume “has stood since it was presented,…not really believed but withstanding all attempts to overturn it. The continuing failure suggests that it might be actually correct.”

Arguing that the problem of induction is a real philosophical issue, the focus then turns to those established responses that have acknowledged the insolvability of Hume’s original exposition of the problem and have attempted to cope with alternative forms of reasoning that address Hume’s concerns. There are two streams of theoretical work that have been particularly influential in this category in the 20th century philosophy and could offer a framework for scientific and practical reasoning: pragmatic induction and critical rationalism (cf. Lange 2011; Vickers 2018).

Both accept Hume’s argument that inductive theories and predictions are rationally unjustifiable and cannot thus be taken as logically valid or truthful claims. The two responses, however, propose different solutions to the original problem, especially from the perspective of predicting or anticipating unexpected futures. Pragmatic induction focuses on a gradualist accommodation of new observations in the context of inductive analysis of probabilities; while critical rationalism proposes a more deductive approach based on the testing of theoretically hypothesized predictions.

Pragmatic vindication of induction was originally articulated by Peirce (1878; Buchler 1955), and later substantially reworked by Reichenbach (1938) and Salmon (1967/2017; 1991). Peirce (1878) accepted Hume’s claim that inductive reasoning cannot be rationally justified. However, following Hume’s view about the inductive habits of mind, he argued that induction can be nevertheless used as a method of approaching truth. In Peirce’s thought, induction is closely related to probability, in a sense that any inductive inference provides a tentative account about the likelihood of a phenomenon appearing in a certain way. Whereas a single exercise of inductive analysis produces a weak theory of suggested probabilities, subsequent empirical observations will over time correct the possible flaws of the initial inferences (Mayo, 2005).

Moreover, according to Peirce, induction can be a valid form of reasoning provided there is a community of inquiry that pursues a tentative inductive theory further by executing further empirical analyses on the issue. Scientific community refines existing inductive theories in part by responding to the emerging anomalies that the prior theories cannot satisfactorily explain. The discovery of instances that deviate from the suggested theoretical regularities motivates a retroductive analysis of alternative conceptual or causal schemes that can better explain the totality of observations (Almeder 2007).

Overall, the pragmatic re-assessment of induction consists of two main pillars. Firstly, inductive inferences are interpreted primarily as probabilities deduced from a finite set of observations. As such, any inductive inference aims to provide provisional theories that suggest tentative generalities with a certain degree of potential error. Theoretical postulations are always fallible, implying that they cannot be validated as universal truths. Yet, even in the absence of certainty of any theoretical claims, induction may be useful as a method in helping us to make sense of and orient to the future. Secondly, initial probabilities deduced from a set of observations can in the longer term approach universally valid laws. Unexpected events encountered during experiences aid in revising tentative theories by way of prompting a search for new, better explanations that will cover a totality of different observations.

The second major response to the problem of induction is associated with the work of Popper (1962; 1971). Principally, Popper (1971) accepts Hume’s view that induction is an irrational form of reasoning and thus not philosophically justifiable. However, he refutes Hume’s conclusion that the limitations of induction lead to a thoroughgoing skepticism. Popper’s argument focuses on the notion of inductive claims as universally valid theories. He notes that Hume’s approach to inductively produced theories emphasizes the generation of positive regularities that are supposed to remain effective also in the future. According to Popper (1971), the positive validity of inductive claims is however not the exclusive way of affirming the truthfulness of theories. Another method is to argue that theories or understandings can be subjected to tests of truthfulness by demonstrating that their predictions are false.

Popper (1971) argues that since inductive claims are essentially arbitrary conjectures that have no necessary grounding outside of the habits of mind, they could be conceptualized as hypothetical propositions. Insofar as theoretical claims are seen as guesses rather than tentative regularities, it is possible to scrutinize these hypotheticals to tests of refutation. Hence, for Popper (1962), the solution to the problem of induction is to bypass the challenge of positively affirming the generalizations of observational data, and instead to treat all claims, inductive or deductive, as hypothetical guesses whose truthfulness can only be scrutinized negatively.

Popper’s position leads to a critical form of reasoning, where the progress towards truth requires a systematic program of falsification. There are at every given time a multitude of theoretical claims produced from empirical and speculative works. In Popper’s (1962) vision, all these theoretical propositions need to be subjected to experimental tests, to assess whether they can be refuted or corroborated. Even if a specific theory survives falsification at some point of time, it cannot be treated as a universally valid truth for future knowledge. Instead, claims that have been successfully corroborated must be subjected to further negative trials. Justification of inductive claims is therefore an ongoing process that may never reach the kind of certainty associated with positive truth claims.

In practical terms, Popper’s (1962) solution to the problem of induction calls for bold theorizing on problems at hand, and, at the same time, for critical reflection or testing of those theoretical hypotheses. At the heart of Popper’s thinking is the admission that there are many competing theories whose truthfulness cannot be justified by past observational inferences. For practical reasoning therefore it is commendable to consider many grand theories of the issue under consideration. For Popper, risky hypothesis entailing unlikely events are more valuable than forecasts projecting a continuation of an inductive pattern. An integral part of the testing of universalizing hypotheses is to deduce so called risky predictions that can be subsequently subjected to experimental tests (Barnes 2005; Zachar 2015).

Risky predictions are postulations of unexpected future events that corroborate or refute the proposed theoretical hypothesis. In practical affairs, this entails a degree of danger based on the assumption that risky predictions concern unlikely yet highly consequential events such as crises or disasters. In societal and organizational spheres, the extreme events favored by Popper represent typically the kinds of futures that we want to avoid for ethical reasons. In these instances, the task of critical rationalism is to detect an extreme event before it develops into a full-blown catastrophe. Signals indicating an impending corroboration of a risky prediction should instead convert into precautionary warnings regarding the measures required to mitigate the approaching crisis (Aven 2006).

## The Two Solutions as Different Temporal Approaches to the Unexpected Events

The main differences between the two responses to the problem of induction can be summarized as follows. Pragmatic vindication of induction sees inductive inferences as tentative beliefs or constructions about the future, especially in the form of empirically generated probabilities. Despite knowledge being in principle fallible, inductive guesses about future are useful insofar as they help to alleviate ignorance and initiate action. Induction however is a continuous process of inquiry, where tentative theories must be refined with the help of further inductive analyses. The process of a successive set of observations and analyses advances our knowledge by offering a self-correcting mechanism for our theoretical reasoning.

In essence, the pragmatic approach to inductive reasoning looks into the future with an eye on the potential anomalies or surprising facts. Unexpected events are not actively predicted in beforehand as it is only when encountering an instance that deviates from the earlier scheme that a re-construction of theory begins. In other words, a pragmatic inductivist is waiting in the present to find future anomalies that provide an impetus to generate better explanations of the phenomenon at hand (Nubiola 2005).

If surprising or anomalistic observations are made, the main task of a pragmatist reasoner is to look backwards to generate a new explanation that accommodates the novel observation into the existing body of inductive inferences (CP 5.189, 1903). This retroductive form of analysis uses identified anomalies to improve our tentative theories in terms of creating understandings that suggest more robust probabilities (Simon 1973).

A critical rationalist, instead, abandons the idea of using induction to produce any kind of positive claims about reality (Popper 1971). The Popperian tradition involves instead a systematic testing of different speculative, grand theories. Inductive hypotheses are not taken as tentative claims that will be refined over time as new surprising facts are encountered.

Instead, critical rationalism thinks reflexively about all coherent theories, empirical or imaginary. Inductively generated understandings are treated similarly to those of more speculative theories. An openness to speculative theories leaves more room to consider radically different perspectives on the future as opposed to the pragmatic method, where sets of empirically deduced probabilities are thought to gradually evolve towards truth in a community of inquiry.

A speculative theory that posits a sudden reversal of past trend is of particular interest to the discussion about crises and disasters, since surprising events typically come to be perceived as unexpected or unforeseen against the horizon of past experiences (Thomas 2012). Unexpected events are typically found bewildering because we are lacking an alternative speculative theory that would interpret the arrival of a surprise as normal or expected.

For a Popperian reasoner, the risky predictions of alternative hypothetical theories are to be taken seriously in our endeavors to cope with the limitations of induction. Established theories and probabilities are best tested using bold or unintuitive predictions suggested in alternative speculative theories. In practical affairs, risky predictions are difficult to implement, but they can be simulated with the help of thought experiments or analogies to related events.

The foregoing discussion could be summarized by noting that in the pragmatic response to the problem of induction, a reasoner is waiting for a new future-in-present to manifest itself in the form of surprising events or cases. Unexpected events are not anticipated before they happen. Once experienced, anomalous observations are used to craft better explanations of past and current events. In essence, a pragmatic inductivist reacts to the possibility of an unexpected event after a surprising incident has occurred.

**Fig. 1:**
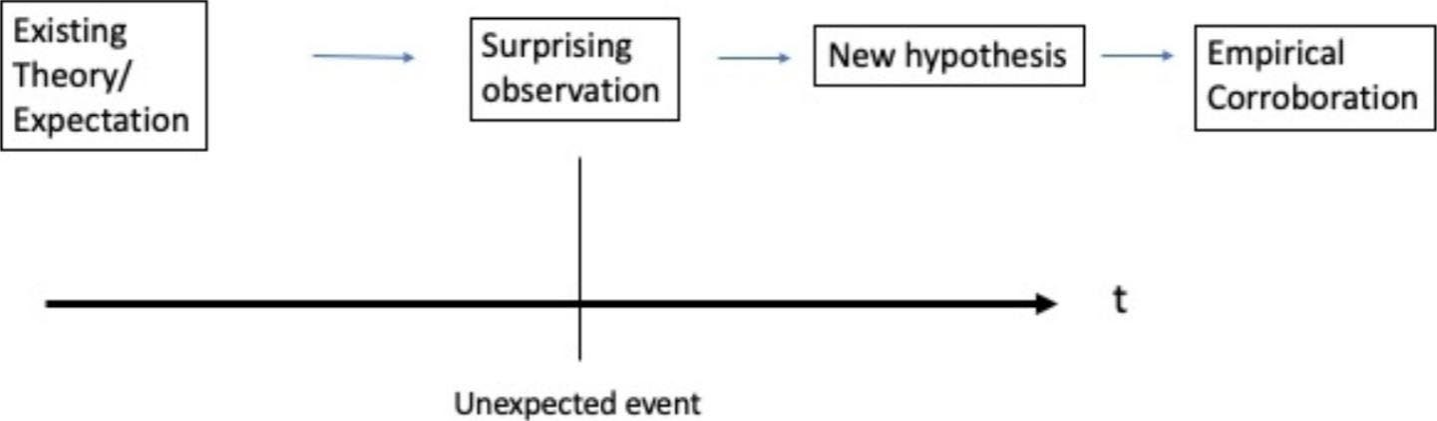
The approach of pragmatic induction (Peirce) on unexpected events

**Fig. 2:**
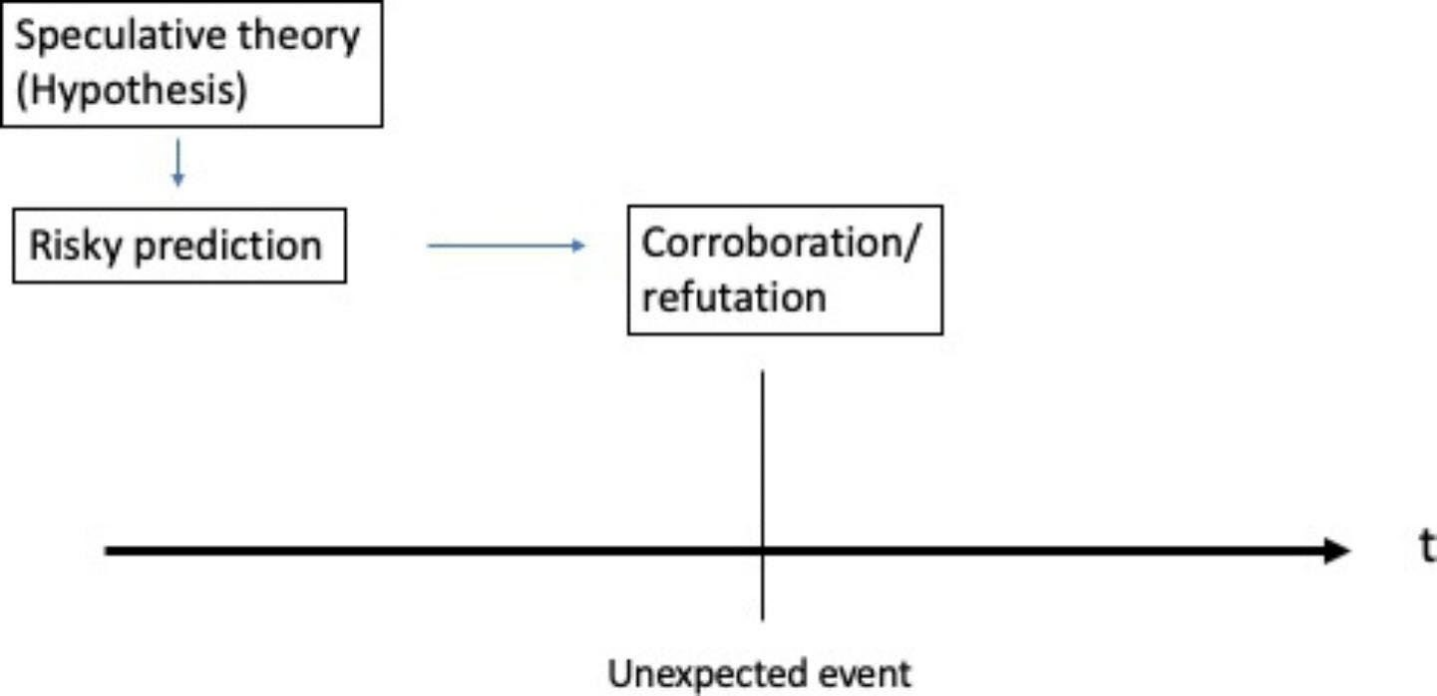
The approach of critical rationalism (Popper) on unexpected events

Critical rationalism, on the other hand, is actively evaluating different theoretical claims before they happen. A Popperian reasoner scrutinizes the future into the test of risky predictions, believing that unconventional or nonintuitive predictions provide the best medium for challenging and potentially falsifying established theories or understandings. In this regard, a critical rationalist reasoner is invested in specifying the risky predictions of hypothesized theories as they may happen in the future. Generally, it could be said that a critical rationalist is looking ahead to several theoretical futures, paying particular attention to the possibility of the materialization of the unexpected events. Figures 1 and 2 condense the different temporal approaches to unexpected events exercised by pragmatic induction and critical rationalism.

## The Predictive Work Related to Covid-19 at the World Health Organization

This section provides a brief overview of the predictive work in the WHO during early 2020. In the weeks following the initial report of a new coronavirus disease in late December 2019, WHO was slow to make a judgement about the severity of the disease. The emergency committee was convened on 22 January 2020 (World Health Organization, 2020a) but was unable to declare a public health emergency of international concern until a week later (World Health Organization, 2020c). Furthermore, the organization delayed naming the outbreak of Covid-19 a “pandemic”, which it finally did on 11 March (World Health Organization, 2020e). The hesitation of WHO to communicate that the new disease was a severe public health risk probably contributed TO SOME DEGREE to the weak preventive response around the globe. An investigation on the early stages of the pandemic concluded afterwards that there was a possibility for more rapid action and an escalation of response following the emerging information about the spread of the virus (Independent Panel for Pandemic Preparedness and Response, 2021).

Available material suggests that the risk officials were hesitant to declare a biomedical threat because there was not enough substantial information to verify the initial observations. WHO experts saw that they needed more local data to evaluate the pandemic risk of the new Covid-19 VIRUS. A leading official of the WHO team for example complained in a meeting that “we’re going on very minimal information”, suggesting that it was the amount of evidential information that was hampering the risk judgement within the organization (Associated Press, 2020). Epidemiologists were deliberating whether to announce some sort of policy proposal under uncertainty or to wait for further verification of the initial reports as was the normal procedure. Eventually, the organization decided to delay announcing higher biomedical threat until more data was available.

The ambiguity of WHO’s reasoning in January and February 2020 was reflected in the conflicting messages the organization provided to the public. On January 23, the General-Director noted in his press conference that WHO’s risk assessment is that the outbreak is a very high risk in China, and a high risk regionally and globally, yet he declined to announce a formal risk warning more generally (World Health Organization, 2020b). On January 30, WHO declared Covid-19 as an international public health risk, but also noted that local decisions in countries need to be evidence-based and consistent. The organization did not advocate measures that would unnecessarily interfere with international travel and trade (World Health Organization, 2020c). On February 15, the General-Director said in an address that the disease is still primarily a local concern for China, but, nevertheless, that there were concerns that the epidemic can spread globally. Without solid evidence, the director was compelled to state that at that moment, it was impossible to predict which direction this epidemic will take. His comments also emphasized that any response should be guided by evidence and public health priorities (World Health Organization, 2020d).

## Evaluating the Style of Predictive Reasoning at WHO

Given the outline of the predictive work at the WHO, it is now possible to evaluate the form of reasoning in contrast to the two responses to the problem of induction. The style of reasoning at WHO in the early weeks of the Covid-19 epidemic highlighted the necessity to obtain enough information to build a detailed picture of the nature of the viral disease. The assumption was that to be able to recommend effective containment practices, there needs to be a sufficiently accurate understanding of the behavior of the new coronavirus and its health effects. WHO wanted to give a more sophisticated policy advice to national health officials than that of a general warning of a potential global pandemic. In press conference comments, experts often voiced their hesitation against large scale containment measures like air travel restrictions or border closures. For example in February 17, a leading official stated that : “…measures should be taken proportional to the situation based on public health, science and evidence, and blanket measures may not help so that’s what we’re trying to say.” (World Health Organization, 2020f).

In terms of the two philosophical responses to the problem of induction, the style of reasoning employed at WHO followed more closely the method of pragmatic induction. The first surprising observations were treated as calls for a revision of the prevailing knowledge regarding the coronavirus infections. There were signs of a new type of disease that differed from the previous SARS and MERS epidemics. Yet the knowledge from the earlier coronavirus diseases and even from influenza epidemics was thought to provide a workable background understanding that could be built upon to arrive at a theory of the new disease and its characteristics.

As leading epidemiological forecasters have argued, it was important for the public health experts to gather enough observational data to construct a sophisticated theory of the disease and its effective containment and treatment (Ioannidis et al. 2020). While there was an inductive theory of coronavirus diseases built based on the previous SARS and MERS epidemics, the novel characteristics of Sars-CoV-2 meant that the prevailing understanding needed to be substantially revised for the release of any evidence-based policy advice from WHO (Liu et al. 2020). The argument was that scientifically based policy guidance must be designed with a reference to the empirically grounded probabilities and mechanisms. For example, containment activities such as lockdowns should be calibrated to the known characteristics of the disease to avoid rash measures that can turn out to be disproportionate to the actual protective aims of the activities.

The shortcoming of the pragmatic approach is that it does not have prior coherent theories or hypotheses to be instantly activated upon the happening or emergence of an unexpected event. Pragmatic induction views anomalies as occasions for revising the existing inductive understanding rather than suggesting a revolutionary shift in our understanding. In the case of the early weeks of the Covid-19 epidemic, WHO was reluctant to declare the novel coronavirus disease an international public health risk or a pandemic, despite that there was a general assumption of a coming disease that would evolve into a major life-threatening pandemic (Cousins 2018). The existing inductive theory was that coronaviruses can cause dangerous diseases, but that the local outbreaks can be contained with informed and targeted public health measures (cf. Michelson 2005).

The main task of the WHO experts in the early phase of the Covid-19 epidemic was to validate the novel characteristics of the new disease with sufficient inductive accuracy. There was concern that without such empirically grounded understanding, public health measure might be misplaced, excessive, or, alternatively, unassertive. Inductive probabilities were too vague to support policy measures. As a result, WHO was cautious in its early advice concerning the more radical preventive measures such as limiting international air travel. It did not want to appear as alarmistic without supporting inductive evidence.

From a critical rationalist perspective, the decision to proceed along the lines of pragmatic reasoning meant that the window of opportunity to launch a series of preventive measures was lost during the early weeks of the epidemic. As for example Taleb et al. (2020) have argued, the inductive approach adapted by the WHO failed to identify the extreme nature of an impending pandemic that would call for swift action based on the early signals. They claim that pandemics are among the events, where there is a need to respond in a determined manner even without detailed empirical evidence. Drastic containment measures taken to avert a crisis are legitimate in these kinds of situations even though they may also result some degree of economic, social, or psychological harm.

A critical rationalist response would have proceeded from a view that a novel, highly transmissible virus can easily cause an existential risk despite its seemingly restricted scope in the initial observational phases. Although the exact source of the public health risk was not fully known in the hypothesis, the general characteristics of an unexpected virus-related outbreak were sufficiently well articulated in the theory to enable a testing of the initial reports against the proposed conjectures (Cousins 2018; World Health Organization, 2019). The main advantage of a critical rationalist approach compared to the pragmatic induction is that there is a considerably shorter link from observations to preventive measures. A single critical observation such as a clinical report from the informed front-line physicians can be used to motivate cautionary measures like wide-spread lockdowns or restrictions of international travel. A pandemic is a singular event whose implications in terms of the societal risk can be deduced without a process of subsequent inductive analysis.

A major shortcoming, then, from a broadly Popperian perspective, was that WHO was not invested in articulating and being sensitized to risky predictions regarding a devastating pandemic. It could be said that there was insufficient theoretical imagination at the organization to connect early clinical and epidemiolocal signals to an overarching theoretical conjecture about the possibility of a dangerous worldwide pandemic. In a sense, the experts at WHO should have been, in a critical rationalist view, constantly in the lookout for the next major disease, promptly refuting or corroborating the risky predictions based on the informal data fed from their global surveillance system. This would have enabled a swift response in terms of strong calls for drastic preventive measures in countries and regions not yet affected by the disease. In hindsight, a more effective containment strategy could probably have saved hundreds of thousands of lives and could have given time to build up further capabilities to combat the disease (Independent Panel for Pandemic Preparedness and Response, 2021).

## Concluding Discussion

This paper has discussed the limitations of induction in predictive organizational reasoning and evaluated the philosophical alternatives to traditional inductive thought. The problem of induction is a classical philosophical challenge to predictive reasoning, particularly in the context of surprising events that carry considerable risks. According to Hume (2007), making a leap from the past observations to the unobserved future cannot be rationally justified, insofar as future can at any moment introduce a new phenomenon that has not been experienced before. Both deductive and inductive justifications of induction turn out to be inadequate. The problem of induction presents a vexed challenge to predictive reasoning in organizations and institutions, where evidence-based thinking has typically permeated attempts to deduce justifiable forecasts based on experiential wisdom.

This article has discussed two responses to the problem that accept Hume’s critique of induction and aim to develop alternatives to pure inductive reasoning. Pragmatic induction (Peirce 1878; Buchler 1955) is a philosophical tradition that views inductive reasoning as a method of generating probabilities that serve as useful approximations regarding the propensities of the phenomenon under investigation. Inductive analysis is also viewed as gradually developing towards more accurate theoretical explanations within communities of inquiry. Despite its logical limitations, induction is treated as a potent method for generating practically valuable approximations that become more accurate as new analyses and observations are used to “retroductively” revise the tentative theories.

Critical rationalism, on the other hand, abandons the idea of generating positive theories from generalized observations. Associated with the work of Popper (1962, 1971), critical rationalism instead understands theories as speculative guesses that must be rigorously tested with the help of experiments or risky predictions. The strategy of critical rationalism is to subtract specific predictions suggested by theories, and to conduct experiments that serve as critical tests for those theoretical predictions. Furthermore, theories that predict events that are regarded as unexpected or unlikely, are considered stronger than hypotheses, which follow the taken for granted visions. In practical affairs, risky predictions must be partly tested through indirect means, since the aim of practical reasoning is to initiate preventive action before an extreme event is realized in its devastating fullness.

The predictive reasoning taking place within the WHO during the early weeks of the novel coronavirus disease offers a case study on the relative merits and drawbacks of pragmatic induction and critical rationalism. WHO followed a broadly pragmatic inductive approach to predictive reasoning amidst the uncertainty that surrounded the initial reports of a wave of cases of strange pneumonic illness in China. The organization started to revise its existing inductive wisdom about the coronavirus diseases and other viral epidemics as soon as first information about the novel viral illness arrived. However, during the time it took to gather additional observational data about the specifics of the new virus and its health effects, WHO missed a window of opportunity to propose precautionary measures that could have contained the spread of the virus as it was traveling from China to the other parts of the world. The organization was hesitant to declare a major biomedical emergency because it did not have enough observational data to modify its embryonic inductive theories about the disease and its characteristics.

An alternative approach would have deduced that there was at the time sufficient information to reason that the disease constituted a major public health risk at the global scale. This type of reasoning, following the critical rationalist approach, would have detected early clinical reports as sufficient evidence for the practical corroboration of a risky prediction of a threatening pandemic. The theoretical hypothesis according to which a major global pandemic with certain features can emerge at any time would have been validated to the extent that it would have been possible to conclude that there were grounds to take swift action. WHO and the world could have thus initiated drastic preventive measures that would probably have saved thousands of lives and that would have given time to build up a more robust societal response (Independent Panel for Pandemic Preparedness and Response, 2021).

As a conclusion, it can be argued that in the critical first weeks of the mounting crisis, a Popperian approach to predictive reasoning would have been more effective than the method of pragmatic induction. Critical rationalism has a full theory of the outbreak of a global pandemic at hand when confronting the early experiences and observations of a novel viral disease. This speculative theory empowers a swift identification of the risks involved with a seemingly limited epidemic and enables institutions and organizations to move quicker from analysis to action.

On the other hand, once the first preventive measures are taken to protect the public and to buy time to build up capabilities, critical rationalism may be of lesser value compared to the judgements emanating from pragmatic induction (Ioannidis et al. 2020). An inductive analysis generates a more sophisticated understanding about the behavior of the phenomenon. In the case of pandemics, pragmatic induction produces tentative theories about the probabilities of various outcomes as well as on the mechanisms behind different phenomena. Consequently, it provides actors with working knowledge about the effectiveness of different measures that can be used in planning a palette of different procedures as well as balancing the preventive benefits of various actions with the accompanying human and economic costs. As the inductive understanding grows, theoretical insights into the characteristics of the disease and its management are also expected to improve in communities of inquiry.

On a balance, however, the pragmatic appropriation of induction as a method of inquiry suffers from the inherent temporal sluggishness of the inferential procedures when confronted with unexpected events that bring with them significant consequences for societies and institutions. There is an inherent difference between Peirce and Popper regarding the role of a single event in testing (corroborating or refuting) a nascent theory or understanding. As noted, Peirce’s pragmatic scheme suggests that inductive reasoning requires a set of interpretative analyses to approach a higher degree of probability in a community of inquiry (Cheng 1966). Conversely, for Peirce, a single event is not sufficient to move from observations to conclusions. In Popper’s program, instead, a single event has the power to overturn a theoretical conjecture. Popper’s approach to decision-making emphasizes the trial-and-error process of articulating and experimentally testing various theories or predictions, whereas Peirce’s pragmatic induction is best characterized as a self-correcting practice, where a series of observations is required to arrive at a more reliable or complete form of theory (Mayo, 2005).

The focus of this article has been on the contribution of Karl Popper’s (1962; 1971) critical rationalism to predictive reasoning in the situations of unexpected events. The specific case of interest was an evaluation of the styles of inferential thinking during the outbreak of the covid-19 pandemic, especially within the organization of World Health Organization. On a general level, the article adds to the existing corpus of management theoretical studies on Popper’s relevance for organizational research and practice. Previously, Popper’s work has been most prevalent in two distinct areas of management scholarship.

Firstly, his falsificationist method of scientific inquiry has been often recognized in discussions related to philosophy of organizational science. Standard methodology works present Popper as the founder of the hypothetico-deductive model of scientific reasoning, together with a notion that this program is associated with the positivist paradigm of management research (e.g. Easterby-Smith et al. 2002; 51–52; Gill and Johnson 2010; 52–54). In these discussions, Popper’s deductivism is contrasted with the inductive approach that has been garnering more support in the recent years (Woiceshyn and Daellenbach 2018), although scholars such as Moss (2003) have argued for the continued relevance of critical rationalism and falsificationism. A second, albeit more limited, stream of scholarship has engaged with Popper’s social philosophy, in particular his concept of an open society. Armbrüster and Gebert (2002) have for example discussed the use of open society approach for evaluating contemporary patterns of organizing in contemporary companies, whereas Ingrams (2020) has outlined a more concrete open society model of administration to be enacted in governmental affairs.

A third adaptation of Popper’s philosophical work has emphasized his epistemological position as a perspective on organizational and managerial thinking or sense-making. Instead of focusing on research methodology or social philosophy, this stream has underlined the relevance of Popper for a conceptual analysis of organizational or strategic reasoning. Shareef for instance (2007) has argued that a critical rationalist response to the problem of induction offers a possibility to educate future managers into a different cognitive style of reasoning that overcomes the problems of the prevailing Kuhnian pragmatism. In a similar fashion, Faran and Wijnhoven (2012) have emphasized the potential of critical rationalism for correcting the false theoretical beliefs among managers through a method of rigorously challenging the implicit assumptions underlying managerial comprehension. Thomas (2012), instead, uses Popperian philosophy to zoom into a concrete event of managerial reasoning, namely the logic of the financial crisis and its retrospective investigation.

The argument developed in the paper applies Popper’s approach to scientific inferences to the domain of organizational predictive reasoning, thus offering a philosophically informed perspective on issues typically discussed under topics such as managerial cognition, strategic thinking, or organizational sense-making (Grandori 2020). As previous research as noted, management scholarship seems to have eschewed a more systemic exploration of the potential relevance of Karl Popper’s work outside of his well-known contributions to philosophy of science and research methodology (Shareef, 2007; Ormerod 2009). Further research could continue the exploration of the relevance of Popper’s critical rationalism as a distinct style of reasoning in practical and organizational contexts.

Finally, beyond the implications of various facets of Popper’s philosophy for management scholarship, the article has underlined the practical issues related to Hume’s problem of induction. Organizations and institutions frequently face unexpected events emanating from their broader environment (Weick and Sutcliffe 2015). The argument developed here implies that resorting to inductive reasoning may worsen the capability to detect and respond to unusual events, since induction typically assumes a degree of continuity between the past experiences and future incidents. Relying on “evidence-based” thinking undermines the real possibility that patterns of reality as inductively inferred can be abruptly disrupted due to an unanticipated happening. Predictions generated by inductive reasoning are not only philosophically unjustifiable but can be also practically defective in situations of sudden surprises. In this regard, “Hume’s problem” is very much “our problem” as well.

## Electronic supplementary material

Below is the link to the electronic supplementary material.

Supplementary Material 1

## References

[CR1] Almeder R (2007). Pragmatism and philosophy of science: A critical survey. International Studies in the Philosophy of Science.

[CR2] Armbrüster T, Gebert D (2002). Uncharted territories of organizational research: The case of Karl Popper’s open society and its enemies. Organization Studies.

[CR3] Associated Press. 2020. China delayed releasing coronavirus info, frustrating WHO. 2 June 2020. https://apnews.com/article/united-nations-health-ap-top-news-virus-outbreak-public-health-3c061794970661042b18d5aeaaed9fae.

[CR4] Aven T (2006). On the precautionary principle, in the context of different perspectives on risk. Risk Management.

[CR5] Barnes EC (2005). Predictivism for pluralists. The British journal for the philosophy of science.

[CR6] Black M (1958). Self-supporting inductive arguments. The Journal of Philosophy.

[CR7] Bonjour L (1986). A Reconsideration of the Problem of Induction. Philosophical Topics.

[CR8] Buchler J (1955). Philosophical Writings of Peirce.

[CR9] Cousins, S. 2018. *WHO hedges its bets: the next global pandemic could be disease X*. 361. Bmj.

[CR10] Cheng CY (1966). Peirce’s Probabilistic Theory of Inductive Validity. Transactions of the Charles S Peirce Society.

[CR11] Cowton C, Zecha G (2003). Doing it right instead of twice: A Popperian approach to management decisions. Philosophy of Management.

[CR12] CP. 1931–1966. The Collected Papers of Charles S. Peirce, 8 vols., ed. by Hartshorne, C, Weiss, P. and Burks, A. W. Cambridge: Harvard University Press. Cited as CP followed by volume and paragraph number.

[CR13] Easterby-Smith MT, Thorpe RR, Lowe A (2002). Management Research: An Introduction.

[CR14] Eccleston-Turner MR, Phelan A, Katz R (2019). Preparing for the next pandemic—the WHO’s global influenza strategy. New England Journal of Medicine.

[CR15] Eisenhardt KM, Graebner ME, Sonenshein S (2016). Grand challenges and inductive methods: Rigor without rigor mortis. Academy of management journal.

[CR16] Faran D, Wijnhoven F (2012). Critical rationalism and the state of unawareness in managers’ theories. Management learning.

[CR17] Gill J, Johnson P (2010). Research Methods for Managers.

[CR18] Grandori A (2020). Black swans and generative resilience. Management and Organization Review.

[CR19] Habermas J (1992). Postmetaphysical thinking.

[CR20] Henderson, L. 2020. The Problem of Induction. The Stanford Encyclopedia of Philosophy (Spring 2020 Edition), Edward N. Zalta (ed.), https://plato.stanford.edu/archives/spr2020/entries/induction-problem/.

[CR21] Howson, C. 2000. *Hume’s Problem: Induction and the Justification of Belief*. Oxford University Press.

[CR22] Hume, D. (1739/ 2007) A Treatise of Human Nature. Oxford: Oxford University Press.

[CR23] Independent Panel for Pandemic Preparedness and Response. 2021. Second report on progress. Evaluation of the international health response to COVID-19. https://theindependentpanel.org/wp-content/uploads/2021/01/Independent-Panel_Second-Report-on-Progress_Final-15-Jan-2021.pdf.

[CR24] Ioannidis JP, Cripps S, Tanner MA (2020). Forecasting for COVID-19 has failed. International journal of forecasting.

[CR25] Ingrams A (2020). Administrative reform and the quest for openness: A Popperian review of open government. Administration & Society.

[CR27] Ioannidis JPA, Cripps S, Tanner MA (2020). Forecasting for COVID-19 has failed. International Journal of Forecasting.

[CR26] Johnsen BC (1972). Black and the inductive justification of induction. Memoir - American Association Of Petroleum Geologists.

[CR28] Ketokivi M, Mantere S (2010). Two strategies for inductive reasoning in organizational research. Academy of management review.

[CR29] Lange, M. 2011. Hume and the Problem of Induction. In: Dov M. Gabbay, Stephan Hartmann, John Woods (Eds.), Handbook of the History of Logic, Vol. 10, 43–91. Elsevier. 10.1016/B978-0-444-52936-7.50002-1.

[CR30] Liu YC, Kuo RL, Shih SR (2020). COVID-19: The first documented coronavirus pandemic in history. Biomedical journal.

[CR31] Locke EA (2007). The case for inductive theory building. Journal of management.

[CR32] March JG, Sutton RI (1997). Crossroads—organizational performance as a dependent variable. Organization science.

[CR33] Michelson ES (2005). Dodging a bullet: WHO, SARS, and the successful management of infectious disease. Bulletin of Science Technology & Society.

[CR34] Mill, J. S. 1843/1906. A system of logic: ratiocinative and inductive : being a connected view of the principles of evidence and the methods of scientific investigation. 8th Edition. London: Longman.

[CR35] Moss MW (2003). Practically useless? Why management theory needs Popper. Philosophy of Management.

[CR36] Norman, J., Y. Bar-Yam, and N. N. Taleb. 2020. Systemic risk of pandemic via novel pathogens—Coronavirus: A note. New England Complex Systems Institute (January 26, 2020).

[CR37] Taleb N,N, Bar-Yam Y, Cirillo P (2020). On single point forecasts for fat-tailed variables. International Journal of Forecasting.

[CR38] Nubiola, J. 2005. Abduction or the Logic of Surprise. Semiotica, 2005(153), 117–130.

[CR39] Ormerod RJ (2009). The history and ideas of critical rationalism: the philosophy of Karl Popper and its implications for OR. Journal of the Operational Research Society.

[CR40] Peirce, C. S. 1878. *The probability of induction. Illustrations of the Logic of Science IV*. vol. 12. 705–718. Popular Science Monthly.

[CR41] Pinson P, Makridakis S (2021). Pandemics and forecasting: The way forward through the Taleb-Ioannidis debate. International Journal of Forecasting.

[CR42] Popper K (1962). Conjectures and Refutations: The Growth of Scientific Knowledge.

[CR43] Popper KR (1971). Conjectural knowledge: my solution of the problem of induction. Revue internationale de Philosophie.

[CR44] Reichenbach H (1938). Experience and prediction: An analysis of the foundations and the structure of knowledge.

[CR45] Salmon WC (1991). Hans Reichenbach’s vindication of induction. Memoir - American Association Of Petroleum Geologists.

[CR46] Salmon WC (1967). The foundations of scientific inference.

[CR47] Salmon WC (1953). The uniformity of nature. Philosophy and Phenomenological Research.

[CR48] Shareff R (2007). Want better business theories? Maybe Karl Popper has the answer. Academy of Management Learning & Education.

[CR49] Simon HA (1973). Does scientific discovery have a logic?. Philosophy of science.

[CR50] Strawson PF (1952). Introduction to logical theory.

[CR51] Tetlock, P., and D. Gardner. 2015. Superforecasting: The Art and Science of Prediction; Crown: New York, NY, USA.

[CR52] Thomas R (2010). What is the relevance of Karl Popper’s critical rationalism to management studies and practice?. Philosophy of Management.

[CR53] Thomas R (2012). The ‘credit crunch’ from a critical rationalist perspective. Philosophy of Management.

[CR54] Tsang EW, Williams JN (2012). Generalization and induction: Misconceptions, clarifications, and a classification of induction. MIS quarterly.

[CR55] Weick KE, Sutcliffe KM (2015). Managing the unexpected: sustained performance in a complex world.

[CR56] Whitehead AN (1929). Process and Reality: An Essay in Cosmology.

[CR57] Vickers, J. 2018. The Problem of Induction, The Stanford Encyclopedia of Philosophy (Spring 2018 Edition), Edward N. Zalta (ed.), https://plato.stanford.edu/archives/spr2018/entries/induction-problem/.

[CR58] Woiceshyn J, Daellenbach U (2018). Evaluating inductive vs deductive research in management studies: Implications for authors, editors, and reviewers. Qualitative Research in Organizations and Management: An International Journal.

[CR59] World Health Organization. 2019. World At Risk. Annual report on global preparedness for health emergencies. World Health Organization. https://apps.who.int/gpmb/assets/annual_report/GPMB_annualreport_2019.pdf.

[CR60] World Health Organization. 2020a. WHO Director-General’s statement on IHR Emergency Committee on Novel Coronavirus. 22 January 2020. https://www.who.int/director-general/speeches/detail/who-director-general-s-statement-on-ihr-emergency-committee-on-novel-coronavirus.

[CR61] World Health Organization. 2020b. WHO Director-General’s statement on the advice of the IHR Emergency Committee on Novel Coronavirus. 23 January 2020. https://www.who.int/director-general/speeches/detail/who-director-general-s-statement-on-the-advice-of-the-ihr-emergency-committee-on-novel-coronavirus.

[CR62] World Health Organization. 2020c. WHO Director-General’s statement on IHR Emergency Committee on Novel Coronavirus (2019-nCoV). 30 January 2020. https://www.who.int/director-general/speeches/detail/who-director-general-s-statement-on-ihr-emergency-committee-on-novel-coronavirus-(2019-ncov).

[CR63] World Health Organization. 2020d. Munich Security Conference. (Speech by the Director-General). 15 February 2020. https://www.who.int/director-general/speeches/detail/munich-security-conference.

[CR64] World Health Organization. 2020e. WHO Director-General’s opening remarks at the media briefing on COVID-19–11 March 2020. 11 March 2020. https://www.who.int/director-general/speeches/detail/who-director-general-s-opening-remarks-at-the-media-briefing-on-Covid-19---11-march-2020.

[CR65] World Health Organization. 2020 f. Coronavirus press conference 17 February, 2020. Transcript.https://www.who.int/docs/default-source/coronaviruse/who-audio-emergencies-coronavirus-full-press-conference-17feb2020-final.pdf?sfvrsn=d033e2c4_0.

[CR66] World Health Organization. 2022. WHO coronavirus (Covid-19) dashboard. https://covid19.who.int. (read 5/6/2022).

[CR67] Zachar P (2015). Popper, Meehl, and progress: The evolving concept of risky test in the science of psychopathology. Psychological Inquiry.

