# Supplementary material for: Popper’s Critical Rationalism as a Response to the Problem of Induction: Predictive Reasoning in the Early Stages of the Covid-19 Epidemic
Source: Philos Manag. 2022 Oct 24;22(1):7–23. doi: 10.1007/s40926-022-00203-6 (PMC9589766; doi:10.1007/s40926-022-00203-6)
Supplement: Supplementary file 1 — Supplementary Material 1 [file 40926_2022_203_MOESM1_ESM.docx]

*Philosophy of management*

**Popper’s critical rationalism as a response to the problem of induction**

Reply to the reviewers

I am grateful for the insightful and encouraging comments. The paper has been moderately revised in response to the feedback and suggestions from the reviewers. Certain relevant references have also been added to support the argument.

Reviewer #1

Thank you for your comments. I have noted your report in the following ways:

*The novelty and theoretical contribution beyond the case of the Covid-19 pandemic

I have added a brief explanation of how this paper fits into the existing Popper scholarship in management studies (p. 21-22). I distinguish three broad domains of application of Popper: philosophy of science (hypothetico-deductivism, falsificationism), social philosophy (open society) and managerial or organizational reasoning (strategic thinking, organizational sense-making etc). Of these, the third category has been relatively empty so far, and it is the area where the current paper aims to contribute, with a philosophical gist.

*Minor technical notes

These are now corrected

Reviewer #2

Thank you for your comments. I have noted your report in the following ways:

*Why these two responses to the problem of induction?

Thank you for pointing out this important issue and referring to Bhaskar’s solution as one of the alternative responses to Hume. I have now incorporated a concise evaluation of the various responses to the problem of induction to the text (p. 7-8). Within the limits of the word count, I try to argue that there have been three kinds of generic responses (attack Hume’s assumptions, circumvent the problem, or acknowledge the problem) to the problem of induction. I then propose, based on some authoritative views, that Hume has so far withstood most of the challenges to the problem. For instance, Bhaskar’s solution seems to imply a sort of Kantian (transcendental) *a priori* position regarding the uniformity of nature that Hume thought would lead us down to a metaphysical rabbit hole.

My argument is that within those that acknowledge the Hume’s problem, one can identify two responses that have attracted most attention among the social scientists: pragmatic induction and Popper/critical rationalism. This can be seen also as the state of affairs in management scholarship: for example, Simon (1973) has defended Peircean induction, while Popperian thinking is typically presented as the prime opponent of the inductive method. I hope this explication is sufficient for the purposes of the article. Obviously, the philosophical discussion itself has been more nuanced and complex than what can be outlined here.

*Risky hypothesis is more valuable for Popper than projecting a continuation of an inductive pattern?

Basically, Popper argues that risky hypotheses are more valuable since they can provide radically new insights into our theoretical or managerial thinking. The method of starting from the previously observed inductive patterns is in the danger of falling into a verificationist or confirmationist fallacy: a reasoner seeks cases that fit into the existing postulated pattern and fails to note the potential negative cases.

*Pragmatic inductivists must fail, because if they need the future anomaly to make a decision today, they are always too late. Yet pragmatists tend to make decisions without full certainty.

This is an important point. Pragmatism generally endorses fallibilism, but Peirce believed that there is a progress towards truth (defined as higher probability). In situations of unexpected events, a Peircean reasoner aims to use the anomaly to create a new explanation of the totality of observations. Anomalies trigger a retroductive search for a better hypothesis. I think the crucial point here is that unexpected events are used for hypothesis generation, not for hypothesis testing. I refer to this at page 20: for Popper, a single case can alter our theoretical insight and lead to action, whereas for Peirce, a single case is not enough to generate a sufficiently correct new understanding. As a sidenote, it might be good to observe that Peirce’s position departs from the pragmatic interpretation of Reichenbach, who thought that induction as such might be the best alternative method that works in predictive reasoning.

*Other details

These are now corrected or edited.

In addition to the example of car breaks, I refer to the idea of March & Sutton (1991): implied causality between specific measures and organizational performance is often based on the habituated beliefs of managers, not on rigorous empirical scrutiny.
